# An Indoor Visual Positioning Method with 3D Coordinates Using Built-In Smartphone Sensors Based on Epipolar Geometry

**DOI:** 10.3390/mi14061097

**Published:** 2023-05-23

**Authors:** Ping Zheng, Danyang Qin, Jianan Bai, Lin Ma

**Affiliations:** 1 Department of Electronic and Communication Engineering, Heilongjiang University, Harbin 150080, China; 2 National Mobile Communications Research Laboratory, Southeast University, Nanjing 210096, China; 3 Department of Electronics and Information Engineering, Harbin Institute of Technology, Harbin 150080, China

**Keywords:** visual positioning, coordinate transformation, pose estimation, epipolar geometry, built-in sensors, indoor localization

## Abstract

In the process of determining positioning point by constructing geometric relations on the basis of the positions and poses obtained from multiple pairs of epipolar geometry, the direction vectors will not converge due to the existence of mixed errors. The existing methods to calculate the coordinates of undetermined points directly map the three-dimensional direction vector to the two-dimensional plane and take the intersection points that may be at infinity as the positioning result. To end this, an indoor visual positioning method with three-dimensional coordinates using built-in smartphone sensors based on epipolar geometry is proposed, which transforms the positioning problem into solving the distance from one point to multiple lines in space. It combines the location information obtained by the accelerometer and magnetometer with visual computing to obtain more accurate coordinates. Experimental results show that this positioning method is not limited to a single feature extraction method when the source range of image retrieval results is poor. It can also achieve relatively stable localization results in different poses. Furthermore, 90% of the positioning errors are lower than 0.58 m, and the average positioning error is less than 0.3 m, meeting the accuracy requirements for user localization in practical applications at a low cost.

## 1. Introduction

The complex and changeable indoor environment brings great difficulties and challenges to indoor positioning technology. Due to factors such as wall occlusion and multipath effects, satellite signals cannot achieve stable positioning results indoors [1]. Positioning methods based on Wi-Fi [2], radiofrequency identification, Bluetooth [3], ultrawideband (UWB) [4], and ZigBee [5] cannot achieve the mature positioning performance of GNSS because their accuracy is affected by problems such as electromagnetic interference, distance limitation, multipath effects, and high costs. The rapid development of the microelectromechanical system (MEMS) inertial measurement unit (IMU) has been widely used in the military, industrial, and civil fields by virtue of its low cost, low power consumption, and small size. It often appears in various integrated navigation systems with unique advantages. In addition, machine vision positioning promoted by artificial intelligence stands out because it does not require additional equipment [6,7,8]. This image-based method makes it possible to achieve good positioning performance in an economical and applicable way [9]. At present, indoor positioning technology based on a fingerprint database is one of the hotspots of visual localization. This method mainly includes two stages. The first is the offline fingerprint database construction stage, i.e., collecting fingerprint information in the indoor environment and recording the corresponding location labels. The second is position estimation in the online stage, i.e., comparing the fingerprint information input with the database so as to obtain the coordinates of the point to be located.

To obtain high-precision positioning results in a low-cost way and ensure the accuracy of dataset construction with cheap and popular equipment, it is necessary to obtain coordinates that can provide strong support for future navigation. Therefore, in the offline dataset construction stage, we consider the image acquisition equipment, acquisition methods, and how to obtain image attitude and position. Using the image and corresponding information obtained in the offline stage, the disadvantages of the current positioning method are studied to establish the necessity of using epipolar geometry for positioning. According to the analysis of the current pose acquisition methods under the epipolar constraint, we determine the implementation of this study. Lastly, the problems existing in other location determination methods are studied to determine the realization route of obtaining the three-dimensional positioning result.

Image fingerprint acquisition devices are divided into three categories, monocular, binocular, and RGB-D cameras that can obtain depth information. The Kinect sensor developed by Microsoft is most widely used to capture depth information. Some studies [10,11,12] took a depth camera as the carrier to acquire indoor color and depth images, and then built a visual positioning framework on the basis of image features and depth information. The binocular camera can obtain the depth data of target points according to the focal length, baseline, and parallax matrix. However, depth and binocular cameras are expensive, significantly reducing their ease of use and universality. With the popularity of smartphone terminals and the reduction in the cost of vision sensors, indoor visual positioning based on monocular images has broader application prospects.

There exist some practical problems indoors, such as a large area with complex and changeable environments. It is an important problem to construct an effective fingerprint database with limited resources on the premise of ensuring localization efficiency and accuracy. Monocular images can be used to build offline databases by shooting videos [13,14,15], constructing landmark feature descriptors [16], and obtaining fingerprint information at reference points [17,18]. When building an offline database, we prefer getting images with accurate positions and poses. The error divergence is fast when only using IMU for navigation; thus, it cannot complete the task of indoor positioning alone. However, the built-in IMU of smartphones can obtain precise pose information, significantly improving the accuracy of dataset construction. Therefore, we took images at fixed reference points to build an accurate offline dataset. The poses calculated by the built-in accelerometer and magnetometer are used as location labels.

The main task of the online stage is to obtain an accurate position. Some scholars are devoted to research on image retrieval technology [19,20], returning the location label of the retrieved image to the user as the positioning result. Although these image retrieval methods gradually achieve higher accuracy, directly using the retrieval results as localization results depends on the image acquisition density of the dataset [21]. Intensive collection points improve the accuracy, but also have the disadvantages of increasing the workload of dataset construction and the number of retrieval operations, as well as reducing the efficiency. The development of depth restoration technology solves the mutual exclusion of acquisition density and database size. Image-based depth estimation methods directly calculate the depth information from the input RGB images [22,23]. There is no need for expensive equipment, providing a broader application space [24]. According to the number of required input images, it can be divided into monocular depth estimation and multi-view depth estimation. Due to the lack of depth clues, monocular depth estimation often needs to obtain information on the basis of perspective projections, shadows, and other environmental assumptions for calculation, which is an ill-posed problem. The multi-view depth estimation method calculates the depth information on the basis of several observed images. Classical methods include structure from motion (SfM), multi-view system (MVS), and triangulation. However, these methods need to obtain the actual coordinates of some feature points, which is a massive requirement in the offline dataset establishment.

In addition, iterative closest point (ICP) is used to solve the device pose estimation problem in 3D. The depth estimation of monocular images can obtain the relative pose from the object coordinate system to the camera coordinate system through perspective-n-point (PnP) and its improved algorithms [25,26,27]. It can solve the pose estimation problem between 3D and 2D when *n* 3D points and their projection positions are known. Both ICP and PnP need to know the actual 3D coordinates of some features. This kind of method has a massive workload in the offline database construction of large buildings, and it is straightforward to introduce random deviation into the final positioning result due to tool and operation errors.

For monocular images, the fundamental matrix is calculated according to the principle of epipolar geometry, so as to obtain the camera position and pose changes in 2D [28]. The existing fundamental matrix estimation methods can be roughly divided into three types: linear estimation based on algebraic error, nonlinear estimation based on iteration, and hypothesis testing strategy. Among them, the linear estimation method includes the traditional normalized eight-point method [29], seven-point method, n-point method, and improved eight-point method. Fischler et al. proposed a random sample consensus (RANSAC) algorithm [30]. It can estimate the parameters of a mathematical model iteratively from a set of observation data containing outlier points, and it is widely used in solving fundamental matrices. With the deepening of the problem, researchers have proposed improved methods based on the inspection strategy least median of squares (LMedS) [31].

After obtaining the pose information, namely, the rotation vector and the translation vector, it is the last step in the positioning process to determine the final coordinates according to the direction vector whose modulus length is unknown and only represents the relative position relation. A geometric relationship composed of multiple direction vectors can be constructed through the pose transformation of a single query image and multiple dataset images. All direction vectors will theoretically intersect at the same point, i.e., the undetermined point where the user is located. However, it is difficult for multiple vectors to converge under the influence of measurement and calculation errors. The authors of [32,33] calculated the distance from each direction vector intersection point to other direction vectors, and returned the coordinates of the intersection point with the minimum distance to the user. The authors of [34] considered the correct matching of the retrieved images. For each intersection point, its distance to each retrieved image was calculated, and the number of correct matching pairs was used as a weight to multiply the distance. Lastly, the minimum sum of weighted distance of the corresponding intersection point was obtained as the positioning coordinates. Although the above methods can obtain the positioning point, they all project the three-dimensional vector to the two-dimensional plane for calculation. They increase intersection points by reducing dimension and losing altitude information. In addition, they all choose the intersection point that may occur at infinity as the final result, which is prone to being wide of the mark.

**Figure 1:**
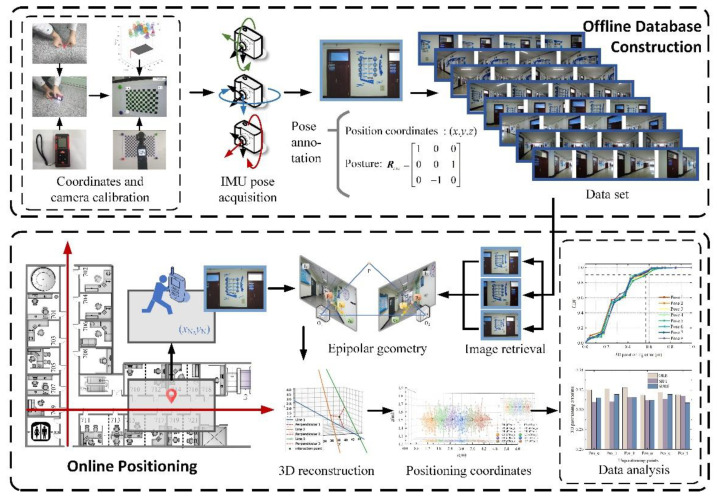
Roadmap of the research.

For this reason, in view of the shortcomings of the existing position determination methods that can only obtain 2D coordinates, taking into account the fact that a single positioning method cannot meet the positioning accuracy requirements of complex and changeable indoor environments, a Three-dimensional reconstruction localization method based on threshold dynamic selection (3D RLM-TDS) is proposed. This method uses an accelerometer and magnetometer to obtain accurate pose labels to construct the dataset. Then, it calculates the relative pose relationship between several images from the dataset with pose labels and the image taken at the point to be located according to the epipolar constraint. In the case that the direction vectors pointing to the undetermined point from the retrieved dataset images cannot converge due to the existence of mixed error, the positioning problem is transformed into solving the minimum distance in 3D space. In this way, we can solve the problem that the direction vector modulus length obtained from the epipolar geometry calculation is unknown, and we can achieve the effect of reconstructing the 3D information in positioning. The roadmap of this paper is shown in Figure 1. The main contributions are as follows:

- An image collection framework is designed with accurate poses to construct the offline database. It uses built-in sensors of a smartphone to obtain relevant data on the basis of Matlab Mobile, and then calculates the Euler angles of the device when shooting images of the dataset.
- In the online stage, an indoor visual positioning method with three-dimensional coordinates using accelerometer and magnetometer is proposed to solve the problems of modulus length loss and only 2D coordinates being obtained in position determination after epipolar geometry. The relative direction of the query image is determined according to the direction vector of the retrieved image pointing to itself. The localization problem is transformed into solving the distance between one point and multiple lines in 3D space to solve the situation that the positioning lines do not converge due to errors.
- A WeChat positioning mini-program mounted on the mobile intelligent terminal is built, and the user’s location is determined in the experimental scene by means of human–computer interaction. The localization error is calculated under different image retrieval schemes, three feature extraction methods, and eight shooting poses, so as to verify the accuracy, robustness, and adaptability of the positioning method proposed in this paper.

## 2. Preliminaries

### 2.1. Feature Extraction and Matching

Image features are the essential characteristics that distinguish a certain type of objects from others, which are the basis and premise of computer vision research such as target recognition, classification, and matching. In the process of feature extraction, features can be divided into global and local features according to the different scopes of extraction. In this paper, the classical blob feature extraction methods scale-invariant feature transform (SIFT) [35] and speeded up robust features (SURF) [36], as well as the corner feature extraction method oriented fast and rotated brief (ORB) [37], are selected to obtain image feature points.

**Figure 2:**
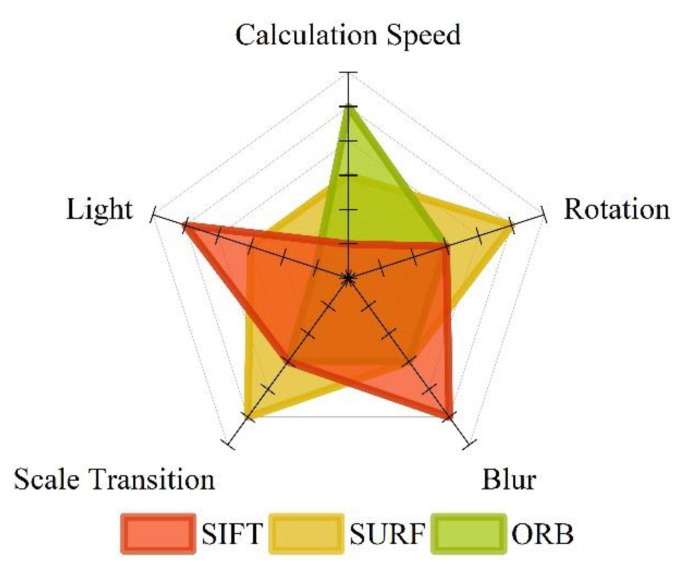
Performance comparison of feature extraction methods.

**Figure 3:**
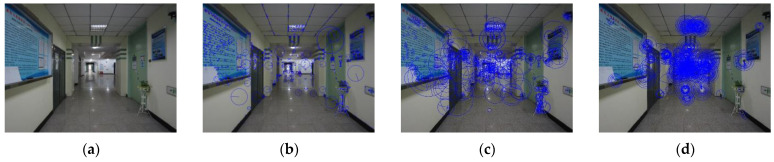
Feature extraction results: (**a**) original; (**b**) SIFT; (**c**) SURF; (**d**) ORB.

The calculation speed, robustness, and other performance comparisons of each feature extraction method are shown in Figure 2, and the corresponding extraction results are shown in Figure 3. The ORB method can achieve faster processing results in situations with high real-time requirements [38,39]. In the case of more illumination and blur, the SIFT algorithm can obtain more accurate feature points. In the case of rotation, scale, and viewing angle changes, SURF can be used to extract blobs more quickly and accurately. Therefore, the above three feature extraction methods have typical characteristics, can resist a variety of interference conditions, and meet the verification conditions of positioning performance.

Feature matching is used to pair the feature points in two images according to a certain similarity standard. Common matching algorithms include brute force (BF) and fast library for approximate nearest neighbors (FLANN). Because BF tries everything, it always finds the best match. FLANN proposed by Muja et al. is an approximation method, which screens the best matching feature points according to the ratio of nearest neighbor to second nearest neighbor. The result found is an approximate nearest neighbor match; hence, it runs more efficiently.

**Figure 4:**
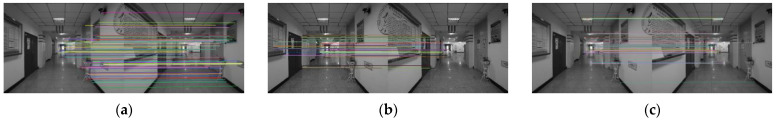
Feature matching results: (**a**) SIFT; (**b**) SURF; (**c**) ORB.

Three feature extraction methods described in Section 2.1 are used to extract images of two scenes, and then FLANN method is used to achieve matching. The corresponding matching results are shown in Figure 4. All three methods can extract a large number of feature points and achieve a high correct matching rate. Since the positioning method is oriented to practical application scenes, in addition to the accuracy, the calculation speed greatly affects the user’s experience. Therefore, the ORB method is mainly selected to obtain matching feature pairs in the subsequent calculation process.

### 2.2. Camera Calibration Principle and Experiments

Camera calibration is used to determine the parameters of the camera imaging geometry model. Calibration accuracy directly affects the accuracy of computer vision applications, especially when the distance is calculated using images. Therefore, it is an essential step to achieve visual positioning.

Camera calibration technologies can be divided into the traditional camera calibration method [40] and self-calibration method [41]. The traditional camera calibration method is time-consuming and labor-intensive, which requires a 3D calibration board with high precision and difficulty to manufacture. Self-calibration does not require calibration objects, but it is not accurate when directly calibrating the camera while only relying on the relationship between the corresponding points of multiple images. Zhang’s calibration method is a compromise between them [42]. Considering the calibration cost, this method does not need to make a refined calibration board, and it has the characteristics of flexible operation and high robustness, thus becoming the most widely used method.

**Figure 5:**
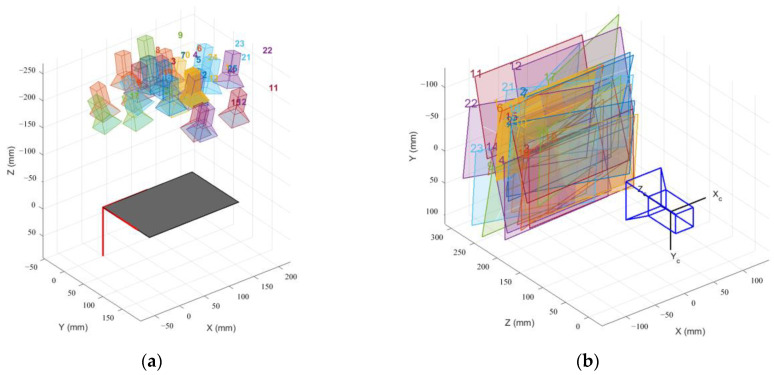
The positional relationship between the camera and the calibration board: (**a**) stationary board; (**b**) stationary camera. Each image is numbered while being taken.

The camera calibration experiment used a 13 × 9 checkerboard, each of which was 15 × 15 mm. Two groups of photos were taken in the experiment, with 25 photos in each group, from the front, left, and right, looking down, and looking up. When taking photos in each direction, the checkerboard calibration board was located at the top left, bottom left, top right, bottom right, and center of the imaging plane, ensuring that the minimum angle between the camera lens and the image plane did not exceed 45°. According to Zhang’s calibration method, images could be taken by means of a stationary image and moving camera or a moving image and stationary camera. The positions between the camera and the board in the experiment are shown in Figure 5.

**Figure 6:**
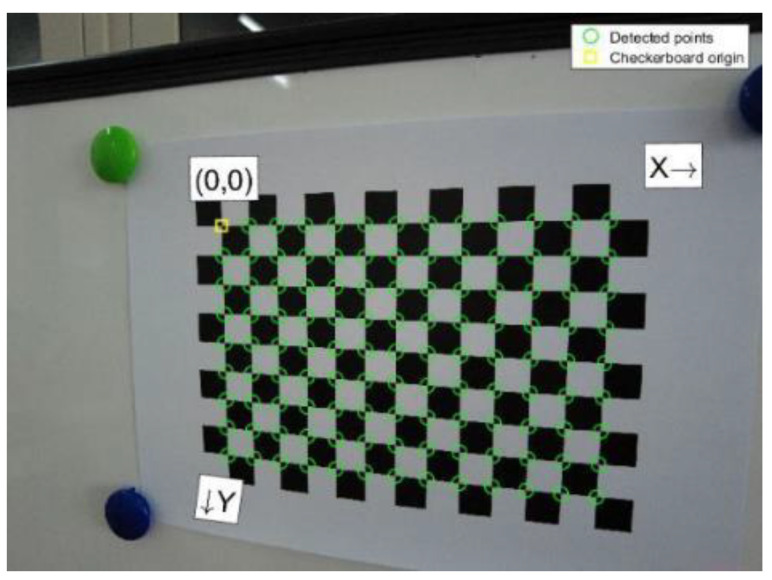
Corner extraction for camera calibration.

**Figure 7:**
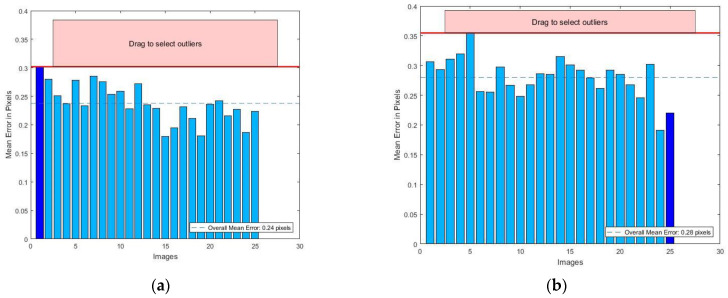
Reprojection error: (**a**) stationary board; (**b**) stationary camera.

Matlab R2016a was used for camera calibration, and the checkerboard after corner extraction is shown in Figure 6. A lower reprojection error indicates a better result. An error lower than 0.5 can meets the accuracy requirement of camera calibration. The average reprojection errors of the two groups were 0.238 and 0.28, as shown in Figure 7, both of which met the requirements. Lastly, the internal parameters obtained from the group with smaller reprojection errors were selected for subsequent experiments, and the corresponding parameters of each group are shown in Table 1.

## 3. Epipolar Geometry 3D Information Reconstruction

### 3.1. Offiline Dataset Construction

#### 3.1.1. Dataset Construction

**Figure 8:**
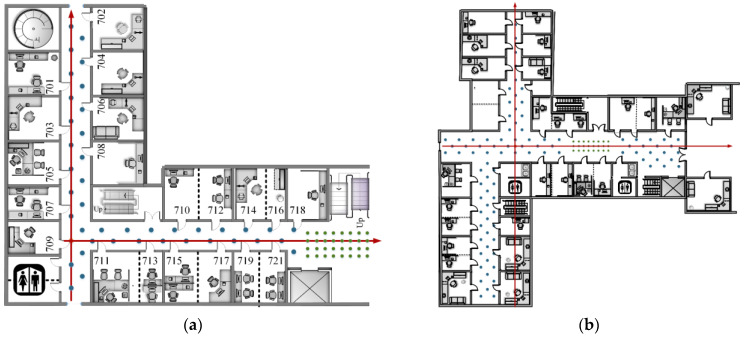
Local maps of the experimental scenes: (**a**) laboratory building; (**b**) teaching building. The numbers in the image are room numbers such as 709, 711 and so on.

In order to verify the performance of the localization method and avoid the particularity of a single experimental environment, the experimental dataset consists of two parts. The local maps of each experimental space are shown in Figure 8. The interval between reference points in the same horizontal direction is 3.6 m, as shown by the blue circle in the figure. Some reference points are added for performance verification in Section 4.2 and 4.3. As indicated by the green circles in the Figure 8, the spacing between them is 0.6 m. The datasets of these two experimental environments meet the needs of positioning and multi-performance verification in practical applications. The reasons for the selection are elaborated in Section 4.1.

**Figure 9:**
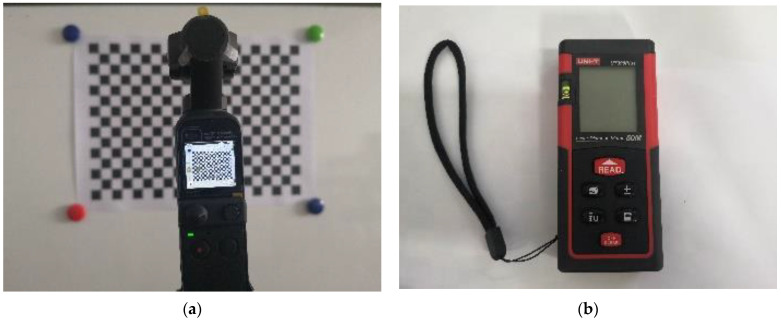
Experimental facilities: (**a**) DJI Pocket2; (**b**) laser rangefinder.

DJI equipment was used to take photos with model DJI Pocket2, shown in Figure 9a. In addition, the precise distance was obtained with a handheld laser rangefinder, shown in Figure 9b, whose maximum range is 50 m, and whose measurement accuracy is ±(1.5 mm + d × 5/10^5^).

#### 3.1.2. Pose Acquisition of Dataset

Each image in the dataset needs to be annotated with the camera pose at the time of shooting for coordinate transformation in the subsequent positioning process. Since rough observations cannot guarantee the accuracy of the data, the built-in sensors were used for precise pose determination when constructing the dataset.

In the process of attitude angle calculation, the rotation order of coordinate axes affects the final coordinate system orientation. In this paper, the roll–pitch–yaw (RPY) method was selected for attitude angle calculation; that is, the rotation order of the axis is *Z*–*Y*–*X*. We used Matlab Mobile to get the rotation angle of the axis according to the information picked up by the accelerometer and magnetometer. When rotating around the *X*-axis *Y*-axis, and *Z*-axis of the inertial measurement unit (IMU), the changed angle is called roll, pitch and yaw, and the rotation angle is *α*, *β*, and *γ* respectively. The corresponding rotation matrix is shown in Equation (1) [43].

(1) $$R_{cw}=R_{x}\left(\alpha \right)R_{y}\left(\beta \right)R_{z}\left(\gamma \right),$$

where ***R***_cw_ represents the rotation matrix that the camera converts from the world coordinate system to the camera coordinate system. ***R***(*α*), ***R***(*β*), and ***R***(*γ*) are shown in Equations (2)–(4), respectively.

(2) $$R_{x}\left(\alpha \right)=\left[\begin{array}{ccc} 1 & 0 & 0\\ 0 & \cos\alpha & \sin\alpha \\ 0 & -\sin\alpha & \cos\alpha \end{array}\right].$$

(3) $$R_{y}\left(\beta \right)=\left[\begin{array}{ccc} \cos\beta & 0 & -\sin\beta \\ 0 & 1 & 0\\ \sin\beta & 0 & \cos\beta \end{array}\right].$$

(4) $$R_{z}\left(\gamma \right)=\left[\begin{array}{ccc} \cos\gamma & \sin\gamma & 0\\ -\sin\gamma & \cos\gamma & 0\\ 0 & 0 & 1 \end{array}\right].$$

Therefore, the rotation matrix in Equation (1) can be further calculated and expressed as Equation (5).

(5) $$R_{cw}=\left[\begin{array}{ccc} \cos\beta \cos\gamma & \cos\beta \sin\gamma & -\sin\beta \\ -\cos\alpha \sin\gamma +\sin\alpha \sin\beta \cos\gamma & \cos\alpha \cos\gamma +\sin\alpha \sin\beta \sin\gamma & \sin\alpha \cos\beta \\ \sin\alpha \sin\gamma +\cos\alpha \sin\beta \cos\gamma & -\sin\alpha \cos\gamma +\cos\alpha \sin\beta \sin\gamma & \cos\alpha \cos\beta \end{array}\right].$$

### 3.2. Online Positioning

#### 3.2.1. Coordinate System and Coordinate Transformation

**Figure 10:**
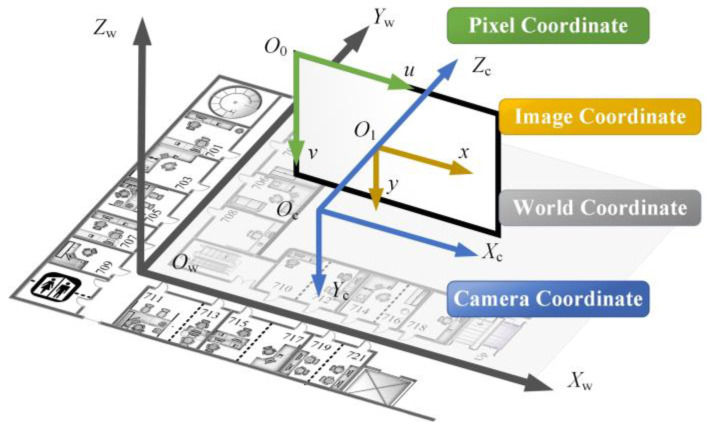
Coordinate system. The room numbers are the same as them in Figure 8.

The positioning process involves the transformation of four coordinate systems. The relative relationship between them is shown in Figure 10. The gray world coordinate system *O*_w_–*X*_w_*Y*_w_*Z*_w_ is a coordinate system artificially set in each positioning scene to facilitate the identification of the position, represented by w. The camera coordinate system *O*_c_–*X*_c_*Y*_c_*Z*_c_ in blue takes the optical central as the origin. The *Z*_c_ axis is the camera’s optical axis, and the *X*_c_ axis and *Y*_c_ axis are parallel to the *X* axis and *Y* axis of the image coordinate system, respectively. The green pixel coordinate system *O*_0_–*uv* takes the upper left corner of the image as the origin and establishes the coordinate system in pixels. *u* and *v* represent the number of columns and rows of a pixel in a digital image, respectively. The yellow image coordinate system *O*_1_–*xy* takes the intersection point between the camera’s optical axis and the image plane as the origin. This coordinate system is established to represent the position of the image in physical units. If the origin of the physical coordinate is *O*_1_(*u*_0_, *v*_0_) in the pixel coordinate system, the relationship between the image coordinate system and the pixel coordinate system is shown in Equation (6).

(6) $$\left\{ \begin{array}{l} u=\frac{x}{dx}+u_{0}\\ v=\frac{y}{dy}+v_{0} \end{array}\right.$$

The unit of *x*/d*x* is pixel. It is expressed in the form of matrix shown in Equation (7).

(7) $$\left[\begin{array}{l} u\\ v\\ 1 \end{array}\right]=\left[\begin{array}{ccc} \frac{1}{dx} & 0 & u_{0}\\ 0 & \frac{1}{dy} & v_{0}\\ 0 & 0 & 0 \end{array}\right]\left[\begin{array}{l} x\\ y\\ 1 \end{array}\right]$$

#### 3.2.2. Position and Pose Acquisition

**Figure 11:**
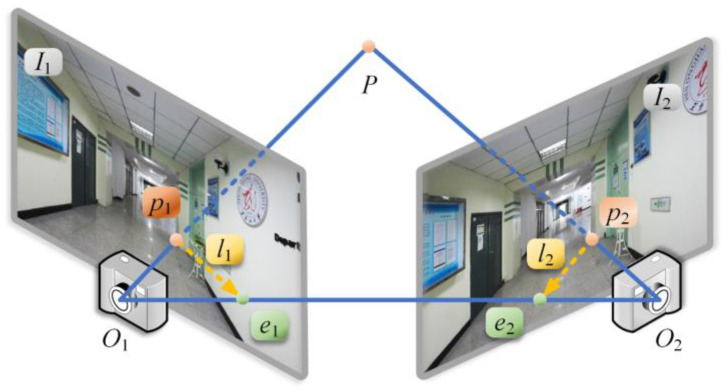
Epipolar geometry relation.

Epipolar geometry describes the geometric information between the two perspective projection images of a single rigid scene, reflecting the pose relationship between the cameras when the two monocular images are taken, and completing the 2D–2D conversion. The epipolar constraint relationship is shown in Figure 11.

The image plane of the camera is *I*_1_ and *I*_2_, and the orange point *P* is a point in the actual space. The projection of this point on the two planes is divided into *p*_1_ and *p*_2_. When using epipolar geometry, *p*_1_ and *p*_2_ are feature pairs with known pixel coordinates corresponding to feature extraction and matching. The line *O*_1_*O*_2_ formed by the center *O*_1_ and *O*_2_ of the two cameras is the baseline, and the plane containing the baseline is the epipolar plane. The baseline intersects each image at the green poles in the figure, denoted by *e*_1_ and *e*_2_, respectively. The lines where the epipolar plane intersects the phase plane are epipolar lines, as shown in yellow *l*_1_ and *l*_2_. The epipolar transformation is represented by ***T***, and ***T***_21_ represents the pose transformation from camera 1 to camera 2, which is composed of the rotation matrix ***R*** and the translation vector ***t***.

Let $P={\left[X_{P},Y_{P},Z_{P}\right]}^{T}$ be the position of point *P* in 3D space; *p*_1_ and *p*_2_ are the corresponding feature pairs of *P*. The transformation between pixel coordinates and world coordinates of two images is shown in Equation (8).

(8) $$Z_{c_{1}}p_{1}=KP,\;Z_{c_{2}}p_{2}=K\left(R_{21}P+t_{21}\right),$$

where ***K*** is the internal parameter of the camera. $Z_{c_{1}}$ and $Z_{c_{2}}$ represent the distance between the image plane of the camera and its optical center when shooting at two positions. ***R***_21_ and ***t***_21_ describe the movement between cameras 1 and 2.

In the process of representing the projection relationship with homogeneous coordinates, the projection plane is usually normalized, i.e., *Z*_c_ = 1, which leads to the loss of length information of the translation vector. In this case, the relationship between pixel coordinates and world coordinates can be obtained as shown in Equation (9).

(9) $$p_{1}=KP,\;p_{2}=K\left(R_{21}P+t_{21}\right).$$

Let the two camera coordinates be $c_{1}=K^{-1}p_{1}$ and $c_{2}=K^{-1}p_{2}$; Equation (10) can then be obtained.

(10) $$c_{1}=P,c_{2}=R_{21}P+t_{21}=R_{21}c_{1}+t_{21}.$$

Both sides of the equation can be cross-multiplied by ***t***_21_ at the same time to get Equation (11).

(11) $$t_{21}\times c_{2}=t_{21}\times R_{21}c_{1}+t_{21}\times t_{21}.$$

As $t_{21}\times t_{21}=\left|t_{21}\right|\left|t_{21}\right|\sin\left(0^{\circ }\right)$, Equation (11) can be simplified.

(12) $$t_{21}\times c_{2}=t_{21}\times R_{21}c_{1}.$$

Since $c_{2}^{T}\cdot c_{2}=0$, Equation (13) can be obtained.

(13) $$c_{2}^{T}\cdot t_{21}\times R_{21}c_{1}=0.$$

The cross-product of vectors is used to calculate their outer product. The cross-product is a vector whose direction is perpendicular to the original two vectors. It is the area of a quadrilateral of two vectors. The cross-product operation of vectors ***a*** and ***b*** can be expressed as Equation (14) [44].

(14) $$a\times b=\left\|\begin{array}{ccc} e_{1} & e_{2} & e_{3}\\ a_{1} & a_{2} & a_{3}\\ b_{1} & b_{2} & b_{3} \end{array}\right\|=\left[\begin{array}{c} a_{2}b_{3}-a_{3}b_{2}\\ a_{3}b_{1}-a_{1}b_{3}\\ a_{1}b_{2}-a_{2}b_{1} \end{array}\right]=\left[\begin{array}{ccc} 0 & -a_{3} & a_{2}\\ a_{3} & 0 & -a_{1}\\ -a_{2} & a_{1} & 0 \end{array}\right]b≜a^{\wedge }\cdot b.$$

According to Equation (14), the cross-product of two vectors can be expressed as matrix and vector multiplication. We introduce the symbol “^” as the skew-symmetric symbol, and we express vector ***a*** as a skew-symmetric matrix, as shown in Equation (15).

(15) $$a^{\wedge }=\left[\begin{array}{ccc} 0 & -a_{3} & a_{2}\\ a_{3} & 0 & -a_{1}\\ -a_{2} & a_{1} & 0 \end{array}\right].$$

Because of $t_{21}\times R_{21}=t_{21}^{\wedge }\cdot R_{21}$, the epipolar constraint can be obtained, as shown in Equation (16).

(16) $$c_{2}^{T}t_{21}^{\wedge }R_{21}c_{1}=0.$$

Since the derivation of the epipolar constraint is obtained on the normalized plane, we cannot judge the specific distance between the cameras. Equation (14) can also be transformed into a form with camera internal parameters, i.e., $p_{2}^{T}K^{-T}t_{21}^{\wedge }R_{21}K^{-1}p_{1}=0$.

The fundamental matrix and the essential matrix reflect the positional relationship between a point on one image and the corresponding point on another image under the epipolar constraint. The essential matrix is $E=t_{21}^{\wedge }R_{21}$; thus, the epipolar constraint can be expressed as $c_{2}^{T}Ec_{1}=0$. The epipolar constraint can also be expressed as $p_{2}^{T}Fp_{1}=0$ because the fundamental matrix $F=K^{-T}EK^{-1}=K^{-T}t_{21}^{\wedge }R_{21}K^{-1}$.

In the process of calculating the fundamental matrix, the eight-point method, seven-point method, RANSAC algorithm, and LMedS can be used. The eight-point method uses eight randomly selected pairs of matching feature points to construct a system of equations. It solves the fundamental matrix according to the singular value decomposition (SVD) method. Both RANSAC and LMedS algorithms need to go through five steps: random sampling, calculation of model parameters, realization of classification, iterative calculation until the optimal solution meeting the threshold is obtained, and precise optimization of model parameters. The main difference between the two approaches lies in the way the classification is implemented. RANSAC divides the inliers and outliers according to the set threshold. LMedS is used to calculate the deviation of the point set of the model, and then find the median of the deviation for classification. The threshold of RANSAC is easier to determine when it has physical meaning or collective meaning, but difficult to adjust when the threshold does not have characteristics. LMedS can obtain the optimal solution through adaptive iteration and is robust to error matching and external points. Therefore, two methods of RANSAC and LMedS are adopted to calculate the fundamental matrix in the process of calculating the epipolar geometry.

**Figure 12:**
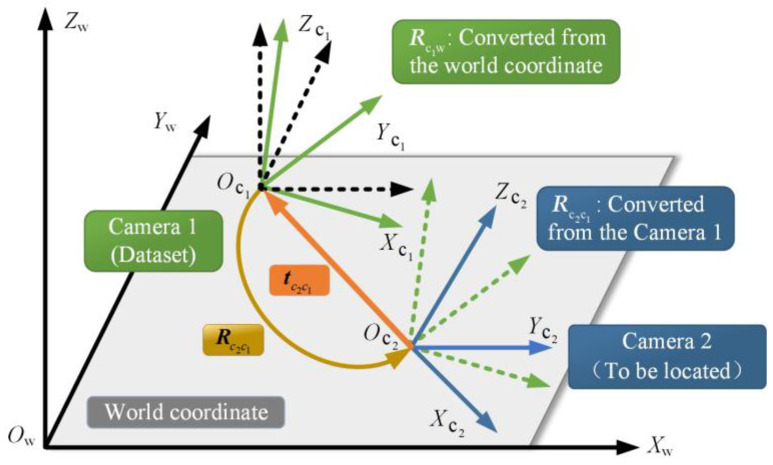
Coordinate system transformation.

The relative pose between the unpositioning node and the dataset images can be obtained using the epipolar geometry, as shown in Figure 12. $O_{c_{1}}-X_{c_{1}}Y_{c_{1}}Z_{c_{1}}$ in green is the coordinate system of camera 1 in the dataset, and $O_{c_{2}}-X_{c_{2}}Y_{c_{2}}Z_{c_{2}}$ is the coordinate system of camera 2 when the images are taken at the point to be located. The transformation relationship between two cameras can be determined through Equation (17), which consists of a rotation matrix $R_{c_{2}c_{1}}$ and a translation vector $t_{c_{2}c_{1}}$.

(17) $$T_{c_{2}c_{1}}=\left[\begin{array}{cc} R_{c_{2}c_{1}} & t_{c_{2}c_{1}}\\ Z & 1 \end{array}\right].$$

The pose of camera 1 is transformed into that of camera 2 through Equation (18).

(18) $$c_{2}=R_{c_{2}c_{1}}c_{1}+t_{c_{2}c_{1}},$$

where $R_{c_{2}c_{1}}$ is the rotation matrix when camera 1 moves to camera 2, and $t_{c_{2}c_{1}}$ is the translation vector from $O_{c_{2}}$ to $O_{c_{1}}$ in camera 2 coordinates. According to the coordinates of the translation vector $t_{c_{2}c_{1}}$ and $O_{c_{2}}$, a straight line passing through $O_{c_{1}}$ and $O_{c_{2}}$ can be represented, and the direction vector of the straight line is $t_{c_{2}c_{1}}$.

Since $t_{c_{2}c_{1}}$ represents the direction vector in the camera 2 coordinate system, it needs to be converted to the world coordinate system during the positioning process. Therefore, the direction vector is reversely transformed according to the pose relationship obtained by the epipolar geometry, as shown in Equation (19), from which the corresponding data can be converted to the camera 1 coordinate system. Then, it is converted to the world coordinate system according to the relationship between the camera 1 coordinate system and the world coordinate system, as shown in Equation (20).

(19) $$c_{2}^{\prime }=R_{c_{1}c_{2}}c_{2}=R_{c_{2}c_{1}}^{-1}c_{2}.$$

(20) $$c_{2}^{\prime \prime }=R_{c_{w}c_{1}}c_{2}^{\prime }=R_{c_{w}c_{1}}R_{c_{2}c_{1}}^{-1}c_{2}=R_{c_{1}c_{w}}^{-1}R_{c_{2}c_{1}}^{-1}c_{2}.$$

Since the rotation matrix is an orthogonal matrix, i.e., $R^{-1}=R^{T}$, Equations (19) and (20) can be further transformed.

(21) $$c_{2}^{\prime }=R_{c_{2}c_{1}}^{T}c_{2}.$$

(22) $$c_{2}^{\prime \prime }=R_{c_{1}c_{w}}^{T}R_{c_{2}c_{1}}^{-1}c_{2}.$$

In the process of constructing the dataset, the position and pose $R_{c_{1}w}$ of the image are marked; thus, the coordinates of the point to be located in the world coordinate system can be obtained.

#### 3.2.3. 3D Information Construction

It is necessary to compare the images captured by the user with those in the dataset. Through image retrieval, the photos are sorted according to the similarity between them, and several results that are closest to the image to be located input by the user in the dataset are returned. If only one image is output, the current retrieval result is identified as the location of the querying user.

When there are two or more retrieved results, the corresponding number of translation vectors can be obtained by calculating the pose between the image to be located and them according to the epipolar constraint. The lack of modulus length of the translation vector makes it impossible to get the specific distance between the dataset images and that taken at the point to be located. The actual position coordinate of the retrieved image is regarded as a point on the straight line, and the translation vector is regarded as the direction vector of the line, so that two lines in the 3D space can be obtained. Theoretically, the intersection of two or more straight lines is the coordinate of the point to be located. However, they do not intersect due to errors during the experiment. Therefore, the localization problem of retrieving two images can be transformed into finding the midpoint coordinates of the minimum distance between two lines. When more images are retrieved, the positioning problem is upgraded to finding the coordinate of a point in 3D space so that the distance between the point and multiple disjoint lines is the smallest.

Compared with obtaining only one image by retrieval, obtaining multiple similar images can increase the credibility of localization. However, it is possible to accumulate errors if too much coordinate calculation is used in positioning. Considering the image collection density and practical application requirements in the construction of the dataset in this paper, it is more appropriate to select the three most similar images as the retrieval results. When the user inputs the image taken at the undetermined node, the three most similar images are obtained through image retrieval, which are annotated with the corresponding position information and poses ***R***_cw_.

Let the coordinates of the point to be located be $P(X_{P},Y_{P},Z_{P})$, and the positions of the three images in the retrieved dataset be *p*_1_$\left(X_{p_{1}},Y_{p_{1}},Z_{p_{1}}\right)$, *p*_2_$\left(X_{p_{2}},Y_{p_{2}},Z_{p_{2}}\right)$, and *p*_3_$\left(X_{p_{3}},Y_{p_{3}},Z_{p_{3}}\right)$, respectively. In the positioning process, *p*_1_, *p*_2_, and *p*_3_ are respectively taken as points on the three lines, and the translation vector ***t*** calculated according to the epipolar geometry is taken as the direction vector of the corresponding line. The symmetrical expressions of the three straight lines are as follows:

(23) $$\left\{ \begin{array}{l} L_{1}:\frac{X-X_{p_{1}}}{t_{x_{1}}}=\frac{Y-Y_{p_{1}}}{t_{y_{1}}}=\frac{Z-Z_{p_{1}}}{t_{z_{1}}}=\alpha_{1}\\ L_{2}:\frac{X-X_{p_{2}}}{t_{x_{2}}}=\frac{Y-Y_{p_{2}}}{t_{y_{2}}}=\frac{Z-Z_{p_{2}}}{t_{z_{2}}}=\alpha_{2}\\ L_{3}:\frac{X-X_{p_{3}}}{t_{x_{3}}}=\frac{Y-Y_{p_{3}}}{t_{y_{3}}}=\frac{Z-Z_{p_{3}}}{t_{z_{3}}}=\alpha_{3} \end{array}\right.,$$

where $t_{x_{1}}$, $t_{y_{1}}$, and $t_{z_{1}}$ respectively correspond to the values of the direction vector ***t*** of *L*_1_ on the *X*, *Y*, and *Z* coordinate axes.

The three lines do not intersect each other due to the error. When the three lines are not parallel to each other, a point can be found in the 3D space such that the sum of the distances from the point to the lines is minimized. The matrix is used to calculate the coordinates of the node.

(24) $$\left\{ \begin{array}{l} X+0\cdot Y+0\cdot Z-t_{x_{1}}\cdot \alpha_{1}-0\cdot \alpha_{2}-0\cdot \alpha_{3}=X_{p_{1}}\\ 0\cdot X+Y+0\cdot Z-t_{y_{1}}\cdot \alpha_{1}-0\cdot \alpha_{2}-0\cdot \alpha_{3}=Y_{p_{1}}\\ 0\cdot X+0\cdot Y+Z-t_{z_{1}}\cdot \alpha_{1}-0\cdot \alpha_{2}-0\cdot \alpha_{3}=Z_{p_{1}}\\ X+0\cdot Y+0\cdot Z-0\cdot \alpha_{1}-t_{x_{2}}\cdot \alpha_{2}-0\cdot \alpha_{3}=X_{p_{2}}\\ 0\cdot X+Y+0\cdot Z-0\cdot \alpha_{1}-t_{y_{2}}\cdot \alpha_{2}-0\cdot \alpha_{3}=Y_{p_{2}}\\ 0\cdot X+0\cdot Y+Z-0\cdot \alpha_{1}-t_{z_{2}}\cdot \alpha_{2}-0\cdot \alpha_{3}=Z_{p_{2}}\\ X+0\cdot Y+0\cdot Z-0\cdot \alpha_{1}-0\cdot \alpha_{2}-t_{x_{3}}\cdot \alpha_{3}=X_{p_{3}}\\ 0\cdot X+Y+0\cdot Z-0\cdot \alpha_{1}-0\cdot \alpha_{2}-t_{y_{3}}\cdot \alpha_{3}=Y_{p_{3}}\\ 0\cdot X+0\cdot Y+Z-0\cdot \alpha_{1}-0\cdot \alpha_{2}-t_{z_{3}}\cdot \alpha_{3}=Z_{p_{3}} \end{array}\right..$$

This can be expressed as Equation (25).

(25) Φ⋅φ=p,

where

(26) $$\Phi =\left[\begin{array}{cccccc} 1 & 0 & 0 & t_{x_{1}} & 0 & 0\\ 0 & 1 & 0 & t_{y_{1}} & 0 & 0\\ 0 & 0 & 1 & t_{z_{1}} & 0 & 0\\ 1 & 0 & 0 & 0 & t_{x_{2}} & 0\\ 0 & 1 & 0 & 0 & t_{y_{2}} & 0\\ 0 & 0 & 1 & 0 & t_{z_{2}} & 0\\ 1 & 0 & 0 & 0 & 0 & t_{x_{3}}\\ 0 & 1 & 0 & 0 & 0 & t_{y_{3}}\\ 0 & 0 & 1 & 0 & 0 & t_{z_{3}} \end{array}\right],\;\varphi =\left[\begin{array}{c} \begin{array}{c} \begin{array}{c} X\\ Y \end{array}\\ Z \end{array}\\ \alpha_{1}\\ \alpha_{2}\\ \alpha_{3} \end{array}\right],\;p=\left[\begin{array}{c} \begin{array}{c} \begin{array}{c} X_{p_{1}}\\ Y_{p_{1}}\\ Z_{p_{1}} \end{array}\\ X_{p_{2}}\\ Y_{p_{2}}\\ Z_{p_{2}} \end{array}\\ X_{p_{3}}\\ Y_{p_{3}}\\ Z_{p_{3}} \end{array}\right].$$

When three lines are parallel to each other, an infinite number of solutions will be obtained to form a line *L_P_*, where the points on the line are the same distance from *L*_1_, *L*_2_, and *L*_3_. When the matrix has a unique solution, the coordinates of the point to be located can be obtained by the least square method. However, when the solution of the equations is not unique (i.e., the rank of the equations is not full), the least square method cannot provide a solution, while the singular value decomposition can effectively solve them. This method can effectively solve the case of both unique solutions and infinite solutions. When the matrix column is full rank, the unique solution can be obtained using both methods.

(27) $$\Phi =U\Omega V^{T}.$$

The general form of Equation (27) is Equation (28).

(28) $$\Phi =\left[u_{1},u_{2},\ldots ,u_{k},u_{k+1},\ldots ,u_{m}\right]\left[\begin{array}{cc} \begin{array}{ccc} \omega_{1} & \cdots & 0\\ \vdots & \ddots & \vdots \\ 0 & \cdots & \omega_{k} \end{array} & Z_{k\left(n-k\right)}\\ Z_{\left(m-k\right)k} & Z_{\left(m-k\right)\left(n-k\right)} \end{array}\right]{\left[v_{1},\cdots ,v_{k},v_{k+1},\cdots ,v_{n}\right]}^{T},$$

where ***Z*** is the zero matrix, *m* = 9, and *n* = 6. The above expression can be further split, as shown in Equation (29).

(29) $$\Phi =\left[u_{1},u_{2},\ldots ,u_{k}\right]\left[\begin{array}{ccc} \omega_{1} & \cdots & 0\\ \vdots & \ddots & \vdots \\ 0 & \cdots & \omega_{k} \end{array}\right]\left[\begin{array}{c} v_{1}^{T}\\ \vdots \\ v_{k}^{T} \end{array}\right]+\left[u_{k+1},\ldots ,u_{m}\right]Z_{\left(m-k\right)\left(n-k\right)}\left[\begin{array}{c} v_{k+1}^{T}\\ \vdots \\ v_{n}^{T} \end{array}\right].$$

The second half of the above equation is ***0***; thus, it can be further simplified to obtain Equation (30).

(30) $$\Phi =\left[u_{1},u_{2},\ldots ,u_{k}\right]\left[\begin{array}{ccc} \omega_{1} & \cdots & 0\\ \vdots & \ddots & \vdots \\ 0 & \cdots & \omega_{k} \end{array}\right]\left[\begin{array}{c} v_{{}_{1}}^{T}\\ \vdots \\ v_{k}^{T} \end{array}\right].$$

To find ***Ω***, the following calculation is required:

(31) $$\Phi^{T}\Phi ={\left(U\Omega V^{T}\right)}^{T}U\Omega V^{T}=V\Omega U^{T}U\Omega V^{T}=V\Omega \Omega V^{T}.$$

If S=ΩΩ, Equation (31) can be transformed into the form of Equation (32).

(32) $$\Phi^{T}\Phi V=VS.$$

Therefore, $\lambda_{i}$ is an eigenvalue of $\Phi^{T}\Phi$, and $v_{i}$ is the corresponding eigenvector of $\lambda_{i}$, which is expressed in Equation (33).

(33) $$\Phi^{T}\Phi v_{i}=\lambda_{i}v_{i},i=1,2,\ldots ,6.$$

Each eigenvalue of the above equation can be calculated according to $\left(\Phi^{T}\Phi -\lambda I\right)x=0$, and the corresponding singular value can be obtained by square root of the eigenvalue, as shown in Equation (34).

(34) $$\omega_{i}=\sqrt{\lambda_{i}},i=1,2,\cdots ,6.$$

According to ΦV=UΩ, the corresponding vector in ***U*** can be obtained as shown in Equation (35).

(35) $$u_{j}=\frac{1}{\omega_{j}}\Phi v_{j},j=1,2,\cdots ,6.$$

The coefficient matrix Φ is decomposed according to the singular value and brought back to the expression Φ⋅φ=p, from which Equation (36) can be obtained.

(36) $$\varphi_{svd}=V_{6}^{T}\cdot \Omega_{6}^{-1}\cdot U_{6}^{T}\cdot p.$$

Therefore, the coordinates of the point to be located are as follows:

(37) $$P(X_{p},Y_{p},Z_{p})=(\varphi_{svd}[1],\varphi_{svd}[2],\varphi_{svd}[3]).$$

The perpendiculars of the three lines are given by Equation (38).

(38) $$\left\{ \begin{array}{l} P_{f\_L_{1}=}p_{1}+t_{1}\cdot \varphi_{svd}[4]\\ P_{f\_L_{2}=}p_{2}+t_{2}\cdot \varphi_{svd}[5]\\ P_{f\_L_{3}=}p_{3}+t_{3}\cdot \varphi_{svd}[6] \end{array}\right..$$

**Figure 13:**
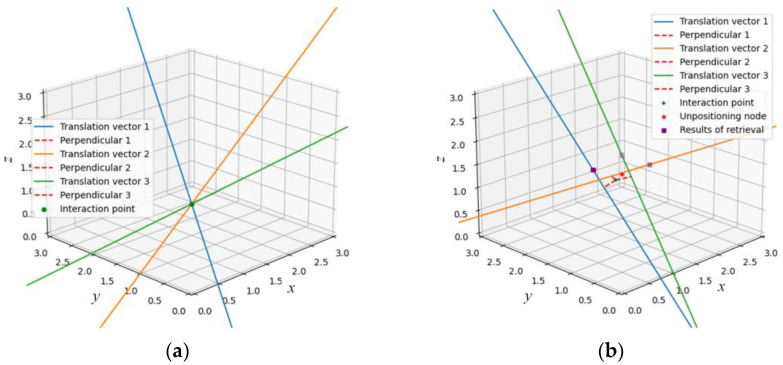
Determination of 3D coordinates: (**a**) ideal error-free case; (**b**) case with errors.

Under the ideal condition without electromagnetic interference, measurement, and calculation errors, the three direction vectors can converge at the undetermined node, as shown in Figure 13a. However, due to the complex electromagnetic environment in the indoor environment, measurement and calculation errors are inevitable, such that the direction vector cannot be effectively converged. Therefore, a three-dimensional reconstruction localization method (3D RML) is proposed to obtain the intersection points and perpendiculars according to Equations (37) and (38), as shown in Figure 13b.

The purple rectangle in the figure is the position of the three most similar images obtained through image retrieval in the world coordinate system. The three lines are the relative direction vectors obtained by epipolar geometry. As can be seen from Figure 13b, 3D RML can find the green intersection point with the minimum distance sum of the three lines when they do not intersect each other as the positioning result. The distance between this positioning coordinate and the real node to be located, shown by the red star, is small.

Considering the different datasets and image acquisition densities used by different researchers, the number of most similar images obtained during retrieval to support positioning is also different. Therefore, the localization method in this paper is extended to retrieve *r* images. Equation (24) can be extended to Equation (39).

(39) $$\left\{ \begin{array}{c} X+0\cdot Y+0\cdot Z-t_{x_{1}}\cdot \alpha_{1}-\cdots -0\cdot \alpha_{k}-\cdots -0\cdot \alpha_{r}=X_{p_{1}}\\ 0\cdot X+Y+0\cdot Z-t_{y_{1}}\cdot \alpha_{1}-\cdots -0\cdot \alpha_{k}-\cdots -0\cdot \alpha_{r}=Y_{p_{1}}\\ 0\cdot X+0\cdot Y+Z-t_{z_{1}}\cdot \alpha_{1}-\cdots -0\cdot \alpha_{k}-\cdots -0\cdot \alpha_{r}=Z_{p_{1}}\\ \vdots \\ X+0\cdot Y+0\cdot Z-0\cdot \alpha_{1}-\cdots -t_{x_{k}}\cdot \alpha_{k}-\cdots -0\cdot \alpha_{r}=X_{p_{k}}\\ 0\cdot X+Y+0\cdot Z-0\cdot \alpha_{1}-\cdots -t_{y_{k}}\cdot \alpha_{k}-\cdots -0\cdot \alpha_{r}=Y_{p_{k}}\\ 0\cdot X+0\cdot Y+Z-0\cdot \alpha_{1}-\cdots -t_{z_{k}}\cdot \alpha_{k}-\cdots -0\cdot \alpha_{r}=Z_{p_{k}}\\ \vdots \\ X+0\cdot Y+0\cdot Z-0\cdot \alpha_{1}-\cdots -0\cdot \alpha_{k}-\cdots -t_{x_{r}}\cdot \alpha_{r}=X_{p_{r}}\\ 0\cdot X+Y+0\cdot Z-0\cdot \alpha_{1}-\cdots -0\cdot \alpha_{k}-\cdots -t_{y_{r}}\cdot \alpha_{r}=Y_{p_{r}}\\ 0\cdot X+0\cdot Y+Z-0\cdot \alpha_{1}-\cdots -0\cdot \alpha_{k}-\cdots -t_{z_{r}}\cdot \alpha_{r}=Z_{p_{r}} \end{array}\right..$$

The coordinates of the point to be located can be obtained according to the singular value decomposition, as shown in Equations (40) and (41).

(40) $$\varphi_{svd}=V_{k}^{T}\cdot \Omega_{k}^{-1}\cdot U_{k}^{T}\cdot p.$$

(41) $$P(X_{p},Y_{p},Z_{p})=(\varphi_{svd}[1],\varphi_{svd}[2],\varphi_{svd}[3]).$$

The perpendiculars to the *r* lines are given by Equation (42).

(42) $$\{\begin{array}{c} P_{f\_L_{1}=}p_{1}+t_{1}\cdot \varphi_{svd}[4]\\ \vdots \\ P_{f\_L_{k}=}p_{k}+t_{k}\cdot \varphi_{svd}[k+3]\\ \vdots \\ P_{f\_Lr=}p_{r}+t_{r}\cdot \varphi_{svd}[r+3] \end{array}.$$

#### 3.2.4. The Optimal Threshold Determination

**Figure 14:**
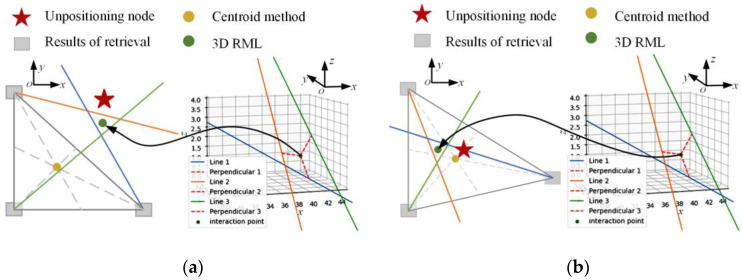
Positioning method switching: (**a**) 3D MSD is more accurate; (**b**) centroid is more accurate.

We constructed a three-dimensional reconstruction localization method on the basis of the above research. This method can determine the 3D coordinates of the camera when the user takes the image at the node to be located in the indoor condition. However, the dataset is constructed from images collected in different areas of the environment, inevitably leading to differences in feature points. The specificity of the image leads to the different support degree of the feature points to the positioning, which results in a significant variance of the positioning error. However, if the coordinate centroid is calculated for most similar images retrieved, the location results can be limited to the vicinity of the image retrieval results. The instability of the search results directly affects the location results, as shown in Figure 14. The gray triangle is the location of the three images obtained by image retrieval, and the yellow circle is the result obtained using the centroid method. At this time, because the retrieval results are on the same side of the real point, the final result is far away from the actual node. Using 3D RLM, the positioning result obtained by calculating the relative pose is closer to the actual position. Although the simple centroid method depends on uniform and accurate image retrieval results, it can limit the result area of the results when the positioning method based on epipolar geometry performs badly. Their complementarity provides a new idea for higher-precision positioning.

Under the condition that the building structure is an irregular rectangle and the room layout is uneven due to different application conditions, a single positioning method is not universal. It cannot meet the needs of accurate indoor positioning. A 3D reconstruction localization method based on threshold dynamic selection (3D RLM-TDS) is proposed for such cases. The positioning method is switched according to an optimal threshold so that the effect of accurate indoor positioning is achieved. When the location result deviates too far from the retrieval results, the centroid method can limit the location result. When the retrieval results are all on the same side of the location point, causing the centroid to be far away from the real coordinates, 3D RLM is used to calculate the final positioning results so as to obtain the coordinate with higher accuracy in continuous and real-time positioning.

**Figure 15:**
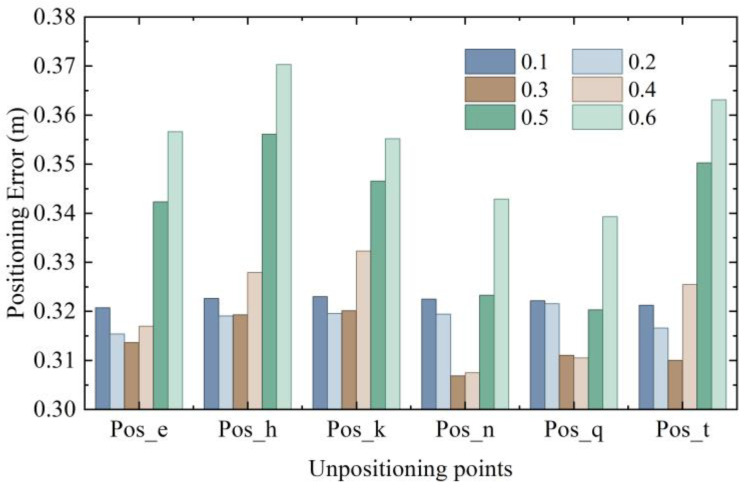
Rough threshold partition.

The measurement points were located according to the epipolar geometry and the centroid method, respectively, and the 3D shortest distance between the positioning coordinates obtained by the two methods was calculated. Taking their gap as the measurement target, six threshold values between 0.1 m and 0.6 m with 0.1 m intervals were selected for a rough test experiment, and six positioning points were estimated. Considering the representative pose and the source range of image retrieval, 2688 data were selected for each group of experiments. The positioning error results under different threshold values are shown in Figure 15.

**Figure 16:**
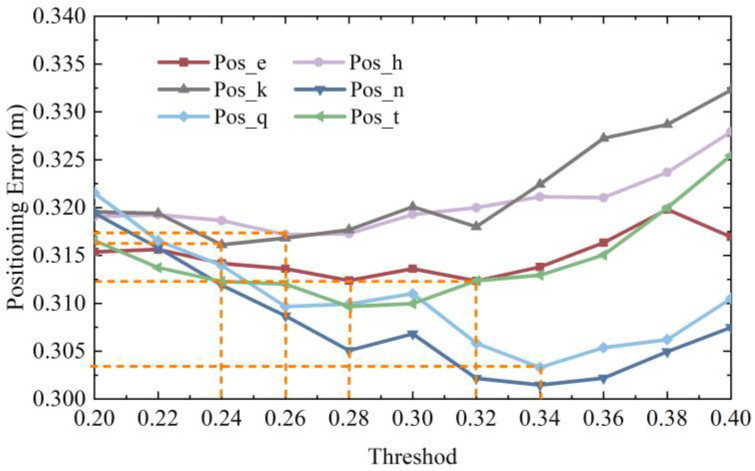
Fine threshold partition.

According to the bar chart, it can be observed that the differences between the coordinates obtained by positioning with different thresholds and the real coordinates generally reached the minimum near the dark-coffee color and the light-coffee color, which means that lower positioning error could be achieved when the threshold value was in the neighborhood of 0.3 to 0.4. In order to obtain a more accurate threshold value, a fine experiment was conducted on the threshold values with an interval of 0.02 within the range of 0.2 to 0.4, and the results are shown in Figure 16.

Although the positioning error from 0.2 to 0.4 fluctuated, the overall trend was concave. The lowest point of error obtained at different thresholds for each node was the intersection point of the orange dotted line in Figure 16. The lowest point of localization error differed because the image features extracted at different positions also differed. Therefore, according to the average value of the threshold when the error of each positioning point was the lowest, it was calculated that, when the image retrieved was eight points around the point to be located, the highest positioning accuracy could be obtained using a threshold of 0.3.

## 4. Performance Verification

### 4.1. Dataset Selection

**Figure 17:**
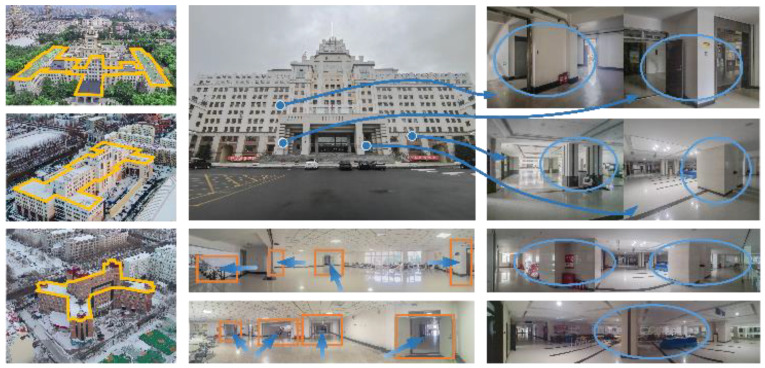
Scenes of dataset.

Large indoor places such as shopping malls, supermarkets, teaching buildings, hospitals, and parking lots are located in complex buildings. As shown in the first column of yellow lines in Figure 17, although the overall structure of the building presents a symmetrical distribution, the external outline is irregular, and the layout of the rooms in the building is also crisscrossed. The corridors, stairwells, and passageways between the buildings are intricate. Therefore, it is necessary to conduct indoor positioning for this kind of environment.

This experiment chose a teaching building as scene Ⅱ, including multimedia classrooms, conference rooms, lecture halls, offices, laboratories, and underground parking lots. The structure of the building is complex, and there are many corridors and intersections. The orange rectangle in the middle of Figure 17 shows the corridors, and the corresponding blue arrow shows their direction. Therefore, it is in line with the building structure that needs indoor positioning. As shown by the blue circle in the last column of Figure 15, the building contains a large number of corners, columns, partitions, etc., which meets the condition of uneven occlusion of the building to the line of sight and the requirements for performance verification of the positioning method. In order to avoid the contingency of a single scene experiment, we selected the laboratory building as scene Ⅰ, which is similar in structure to scene Ⅱ.

In order to verify that the localization method can cope with different image retrieval methods and apply different poses and feature extraction methods, we traversed all possible retrieved images to obtain the final localization result. Eight representative directions were used to represent different poses, and three classical feature extraction methods were selected, covering the extraction of spot and corner features. The positioning results were visualized, and the positioning accuracy was compared.

### 4.2. Positioning Effect

**Figure 18:**
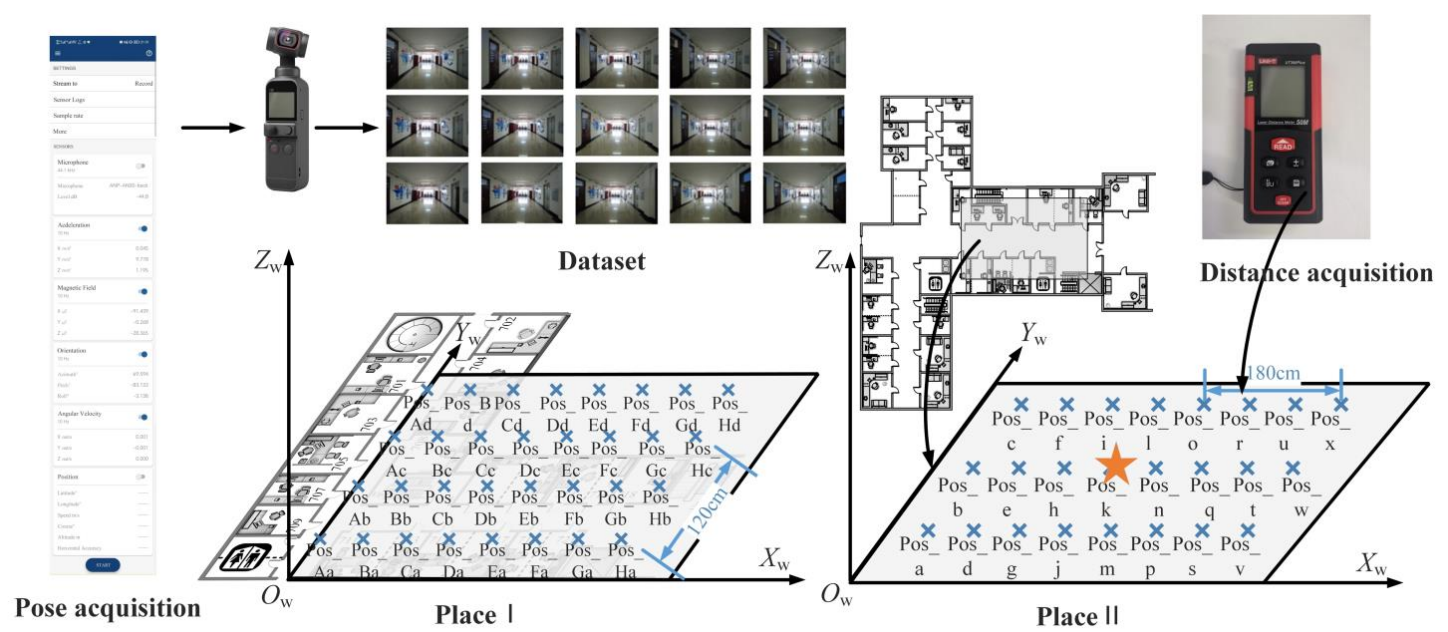
Distribution map of points to be located in experimental scenes.

In order to verify the accuracy of positioning, experiments were carried out on the two scenes mentioned in Section 3.1.1, and the positioning point arrangement is shown in Figure 18.

The corridor in the building was narrow in scene Ⅰ; hence, the square hall in scene Ⅱ was selected as the main test site. Data were collected from five effective shooting directions of 32 test points in this corridor. Considering that the width of the corridor in the actual scene and the positioning performance to be verified need to ensure the diversity of poses, the center line of the corridor was selected as the central axis of the test point in scene Ⅱ, which was expanded at an interval of 0.6 m. The 24 sampling points covered all eight representative directions for image shooting. In order to verify the comprehensive positioning effect and accuracy, scene Ⅱ was taken as an example of the subsequent positioning experiment.

#### 4.2.1. Effect of Different Range of Image Retrieval Results on Localization Accuracy

The purpose of image retrieval is to obtain several images that are most similar to the image to be located. Due to the large camera angle of view, the retrieved results may also be obtained because some local patterns in the angle of view are very similar. However, the distance between the images is far at this time. As shown in Figure 16, the images in the dataset were all taken in the same local area in the same direction. The minimum distance between the images was 0.6 m, and the furthest distance was about 3 m. The similarity between images was too great to ensure that the source range of retrieval results was in a stable state.

Therefore, in order to explore the influence of the distance between the image retrieval result and the point to be located on the positioning accuracy, the experiment was conducted on the range of the result source. Scene Ⅱ was taken as an example, corresponding to the reference points in green in Figure 8b. The position of point Pos_k, represented by an asterisk in Figure 18, is the point to be located, while the remaining points Pos_a to Pos_x are the positions of the images in the dataset.

**Figure 19:**
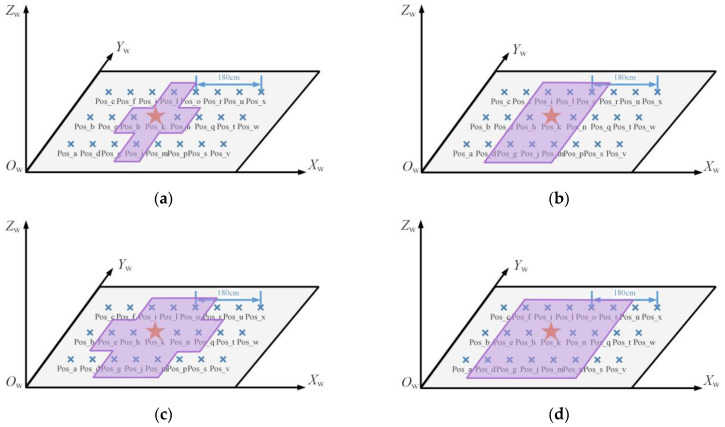
Different ranges of image retrieval results: (**a**) ξ=4; (**b**) ξ=8; (**c**) ξ=10; (**d**) ξ=14. The asterisk represents the point to be located.

The ranges of different sources of image retrieval results are shown in purple in Figure 17. When the retrieval results all came from the four points around Pos_k, namely, Pos_h, Pos_j, Pos_l and Pos_n, as shown in Figure 19a, the distance between the retrieval results and the undetermined site was 0.6 m. When the results came from the surrounding eight, 10, and 14 points, the corresponding source ranges of search results are as shown in Figure 19b–d. The number of surrounding points represents the distance range between the dataset and the node to be located, and the corresponding relationship is shown in Table 2.

**Figure 20:**
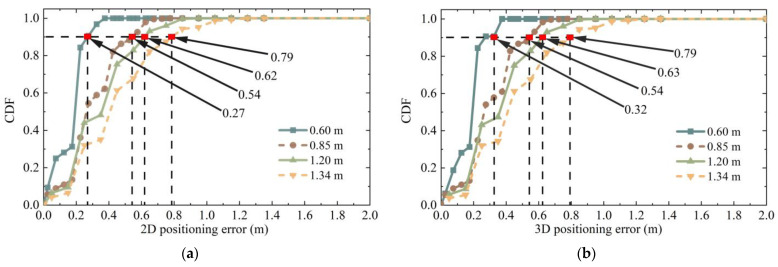
The cumulative distribution function (CDF) of positioning errors under different retrieval results: (**a**) 2D positioning error; (**b**) 3D positioning error.

With different image retrieval results, different source ranges of points were used to determine coordinates, resulting in different final positioning accuracy. When the retrieval results came from eight representative directions of the surrounding ξ nodes, Pos_k was used as the test point for the experiment with the localization method proposed in this paper, and the first three most similar retrieved images were used for localization. Considering that the retrieval results obtained by different retrieval methods differ, all possible results were traversed according to $8\times C_{\xi }^{3}$. When the maximum distance between the retrieved image and the test point was 0.6 m, 0.85 m, 1.2 m, and 1.34 m, the number of data obtained was 32, 448, 960, and 1760, respectively. The cumulative distribution function of the corresponding positioning error is shown in Figure 20. Compared with directly taking the retrieval results as the positioning results, the positioning accuracy improvement rate and other relevant data of the positioning method proposed in this paper are shown in Table 2.

When the source of image retrieval results was within 0.6 m around the node to be located, the 2D error of 90% of nodes could reach 0.27 m using 3D RLM-TDS, improving by 55%. When the distance between the image retrieval result and the actual point to be located was within 1.34 m, and the maximum distance between the retrieved images was 2.68 m, 90% of the 2D and 3D positioning errors were within 0.79 m, improving by 41.04%. Among the four groups of experiments, the second one had the slightest improvement in positioning accuracy at only 36.47%.

In order to verify that the positioning method proposed in this paper could achieve a higher positioning accuracy, the conditions that led the positioning accuracy to present the lowest state were selected in the subsequent positioning experiments. That is to say, the image retrieval range was eight points around the node to be located, and the distance between the retrieval result and the point to be located was 0.85 m. If, under these conditions, 3D RLM-TDS can resist the influence of the two conditions to be verified and present stable positioning accuracy, then it can achieve higher localization accuracy when the retrieval results show the other three groups in better states.

#### 4.2.2. Visualization of Positioning Effect

**Figure 21:**
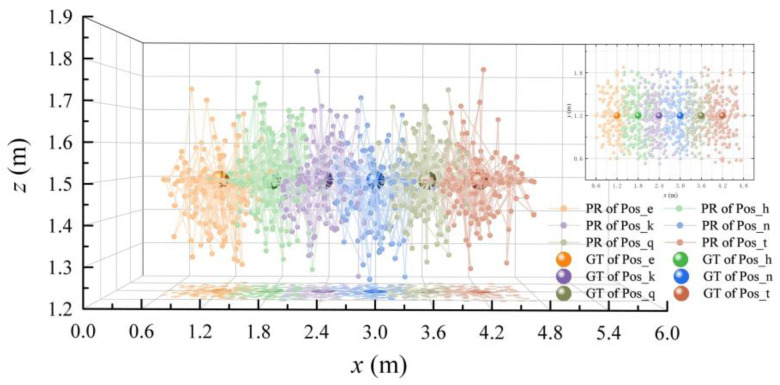
Visualization of 2D and 3D positioning results.

In order to observe the localization experimental results of the proposed method in detail, scene Ⅱ was taken as an example to locate the six points Pos_e, Pos_h, Pos_k, Pos_n, Pos_q, and Pos_t, and the positioning results were visualized. According to the reasons mentioned in Section 4.2.1, eight known points near the test node were selected as the subsequent positioning sources. Three points were randomly selected for epipolar geometry pose calculation. According to $C_{8}^{3}$, there were 56 groups of experimental data in total. The above verification was carried out on the images of the six points and eight directions; thus, 6 × 56 × 8 = 2688 positioning results were obtained. The 2D and 3D positioning results are shown in Figure 21.

The positions of the big ball in Figure 21 represent the ground truth of the six test points, and the small balls with the same color of each test point represent the positioning result of the test point using 3D RLM-TDS. According to the distribution of the positioning points, it can be seen that the positions obtained using the positioning method proposed in this paper were all near the actual nodes, and there were no outliers with a large gap.

### 4.3. Positioning Accuracy Verification

#### 4.3.1. Verification of Positioning Accuracy under Different Positions and Poses

**Figure 22:**
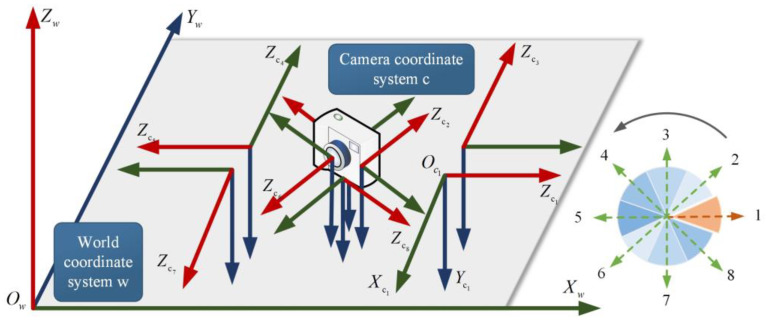
Representative poses.

The localization effect of images taken from different directions was verified in two experimental scenes. The images with eight representative poses are shown in Figure 22. The dataset contained more pose information collected by the built-in smartphone sensors. We took the camera’s optical axis, i.e., the *Z*_c_ axis of the camera coordinate system, consistent with the positive direction of the *X*_w_ axis of the world coordinate system, specified as direction 1. Rotating counterclockwise in 45° increments, the sequence had directions 2–8. The Euler angle and rotation matrix corresponding to the orientation pose are shown in Table 3.

**Figure 23:**
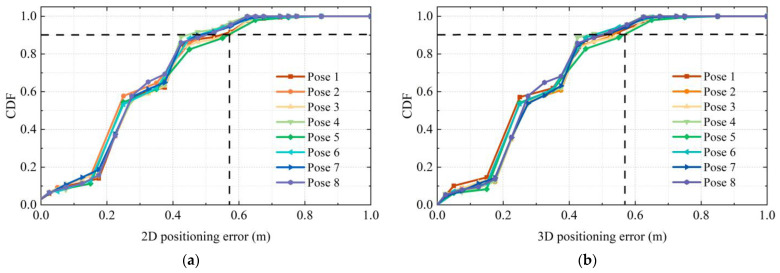
Positioning error under different poses: (**a**) 2D positioning error; (**b**) 3D positioning error.

The 3D RLM-TDS proposed in this paper was used to locate the images taken in eight representative directions, and the 2D and 3D positioning errors are shown in Figure 23. The cumulative distribution trend of positioning errors in all directions was consistent, indicating that the positioning method was not limited to a specific pose. The 2D positioning error and the 3D positioning error showed a uniform trend. Specifically, 90% of the 3D positioning errors were within 0.58 m, verifying the method’s robustness under different poses and meeting the positioning accuracy requirements in practical applications.

#### 4.3.2. Verification of Positioning Accuracy Using Three Feature Extraction Methods

**Figure 24:**
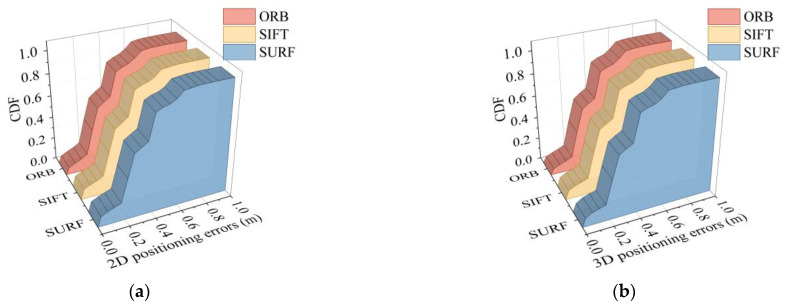
Positioning error under different feature extraction methods: (**a**) 2D positioning error; (**b**) 3D positioning error.

In the experiment, different methods were selected for feature extraction and matching. The poses calculated using the epipolar constraint differed in terms of feature matching pairs. As described in Section 2.1, classical image extraction and matching algorithms include SIFT, SURF, and ORB. Therefore, these methods were used to locate the six points of Pos_e, Pos_h, Pos_k, Pos_n, Pos_q, and Pos_t among the 24 coordinate points in scene Ⅱ. Image retrieval was derived from a dataset of eight points around the point to be located, and each node is verified against the dataset images in eight directions. The corresponding 2D and 3D positioning accuracies are shown in Figure 24.

It can be observed that, no matter which feature extraction method was used for epipolar geometry calculation, the final positioning accuracy showed a uniform trend. It can be seen from the data statistics that 90% of the positioning errors obtained by the three methods were all lower than 0.575 m.

**Figure 25:**
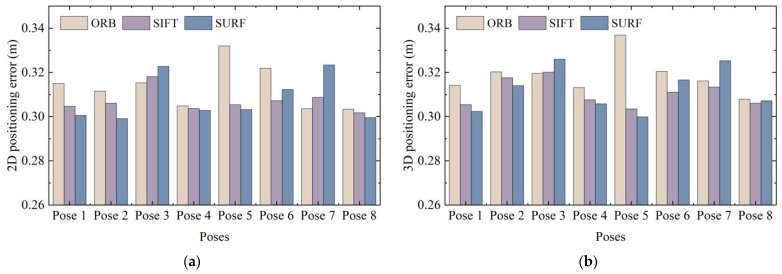
Average positioning error under different poses: (**a**) 2D average positioning error; (**b**) 3D average positioning error.

**Figure 26:**
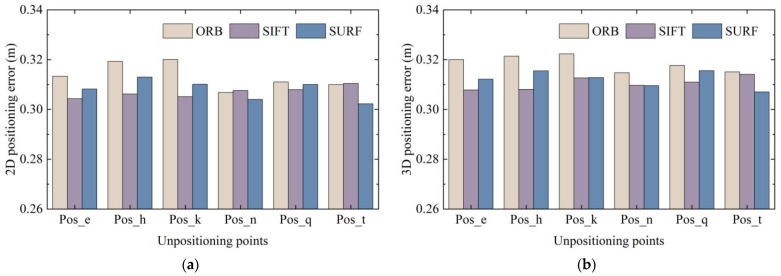
Average positioning error under different feature extraction methods: (**a**) 2D average positioning error; (**b**) 3D average positioning error.

We compared the CDF of positioning error of different pose images and different feature extraction methods. The average positioning error was sorted out to further observe the errors clearly. The average 3D positioning error of the 2688 data obtained using the ORB, SIFT, and SURF methods was 0.3186, 0.3106, and 0.3121 m, respectively. The 2D and 3D average positioning errors of different poses and different positioning points are shown in Figure 25 and Figure 26.

Due to the differences in the patterns and objects in the images captured at different nodes with different poses, the extracted feature points also changed accordingly; thus, the average positioning error obtained also fluctuated. However, it was basically concentrated at about 0.3–0.32 m, showing a relatively stable state. Therefore, 3D RLM-TDS proposed in this paper can cope with different shooting poses, adapt to different feature extraction methods, and resist the influence caused by different source ranges of image retrieval results. It has high robustness and achieves the effect of stably meeting the demand for accuracy in practical positioning applications.

### 4.4. Real-Time Performance Analysis

To verify that the positioning method proposed in this paper can meet the requirements of engineering practice, 100 positioning experiments were carried out, and the time consumption of each involved process was statistically analyzed. In engineering, response times for software interfaces are typically based on certain criteria. When the response time is below 2 s, it is considered a “very attractive” user experience. Users rate a response within 5 s as a “relatively good” experience. However, if the response time is between 5 s and 10 s, it is considered a “bad” experience, and, if no response is received after 10 s, the request is judged to have failed. Therefore, if the time is less than 5 s from when the user uploads the image to when the positioning coordinate is obtained, the positioning system can be considered to be real-time and meet the needs of practical applications.

**Figure 27:**
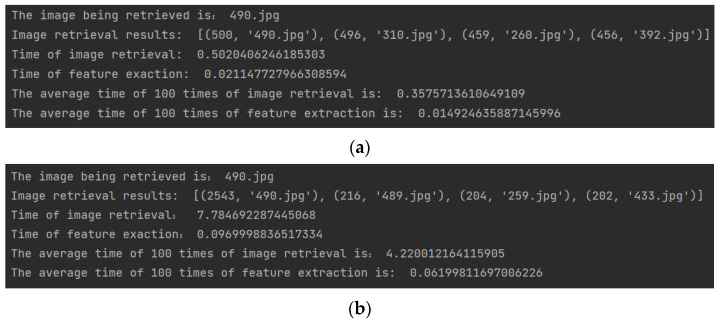
Feature extraction and image retrieval time: (**a**) ORB; (**b**) SURF.

After the user uploads the image, it needs to go through four main steps: feature extraction, dataset image retrieval, relative pose calculation, and 3D RML-TDS location determination. According to the relevant features shown in Figure 2, ORB and SURF, which are relatively efficient, were selected for feature extraction and retrieval to shorten the overall positioning response time. A total of 100 positioning experiments were carried out at different nodes. The image feature extraction and retrieval time were determined, and the average value was calculated. The program operation results are shown in Figure 27.

The experiment showed that the feature extraction time of ORB and SURF was 14.9246 ms and 61.9981 ms, respectively. The time of image retrieval is related to the method, dataset size, and the similarity degree of dataset images. In this experiment, the dataset consisted of 500 images, and the average time of 100 retrievals for ORB and SURF features was 0.3576 s and 4.22 s, respectively. The relative pose calculation process of a pair of images was about 30 ms. We selected the three most similar images to get poses. Therefore, acquiring the three translation vectors required a total of 90 ms. The matrix operation and position switching based on a threshold in 3D RML-TDS consumed a short time, and the acquisition from attitude calculation to final positioning result could be achieved in 100 ms. To sum up, the overall time of feature extraction and image retrieval based on ORB and SURF with 3D RML-TDS for positioning was 472.5246 ms and 4381.9981 ms, respectively. That is, 3D ML-TDS (ORB) and 3D ML-TDS (SURF) could be positioned within 0.5 s and 5 s, respectively, meeting real-time requirements.

### 4.5. Construction of Positioning Mini Program and Comparison of Localization Performance

#### 4.5.1. Performance Comparison of Location Determination Methods

**Figure 28:**
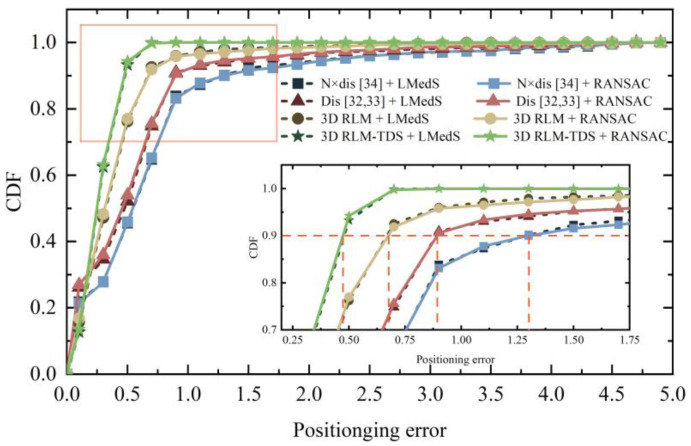
Comparison of localization methods with other studies. The plus sign denotes a combination of different positioning methods and pose acquisition methods.

In order to further verify the performance of the positioning method proposed in this paper, the pose estimation method proposed in [30,31] and the position determination method used in [32,33,34] were selected for combined experiment. In order to unify the preconditions for comparison, the three most similar images obtained from image retrieval were selected for pose determination and positioning point calculation. The cumulative distribution of positioning errors and average positioning errors are shown in Figure 28 and Table 4, respectively. Among them, 3D RLM is the positioning error obtained by solving the minimum distance sum in three-dimensional space without threshold constraints. N × dis is the positioning method considering the quantity of feature matching in [34], and Dis corresponds to [32,33]. The first two items in Table 4 refer to the location obtained by direct image retrieval using ORB and SURF, respectively.

Combining Figure 28 and Table 4, it can be observed that the errors of these four positioning methods were similar when the pose estimation method was changed. Since N × dis and Dis methods map 3D vectors to 2D, large outliers are generated when translation vectors are obtained by LMedS. RANSAC can achieve a more stable positioning situation. However, 3D RLM-TDS does not select the intersection point that may occur at infinity as the positioning result and adds the constraint on the location of the retrieved image; thus, the error obtained is significantly lower than other methods.

#### 4.5.2. Construction of WeChat Positioning Mini Program and Positioning Results

**Figure 29:**
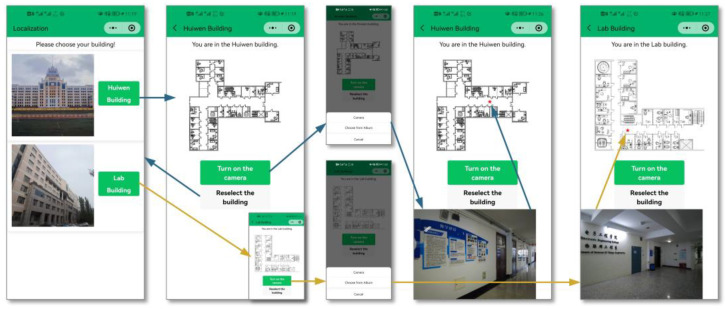
WeChat positioning mini program. Different colored lines correspond to different scenes.

In order to verify the positioning performance of this positioning method in an actual scenario, we developed the WeChat positioning mini program mounted on the mobile smart terminal. It is based on the WeChat developer tools, and the .wxml, .json, .js, and .wxss files were written to build the program interface, as shown in Figure 29. The instruction to retrieve corresponding dataset by SURF is sent according to the building selected by the user. Photos chosen from albums or taken by users are compressed and uploaded to obtain poses by RANSAC. Lastly, the adjusted coordinates calculated by 3D RLM-TDS are transmitted to the mini program for marking.

**Figure 30:**
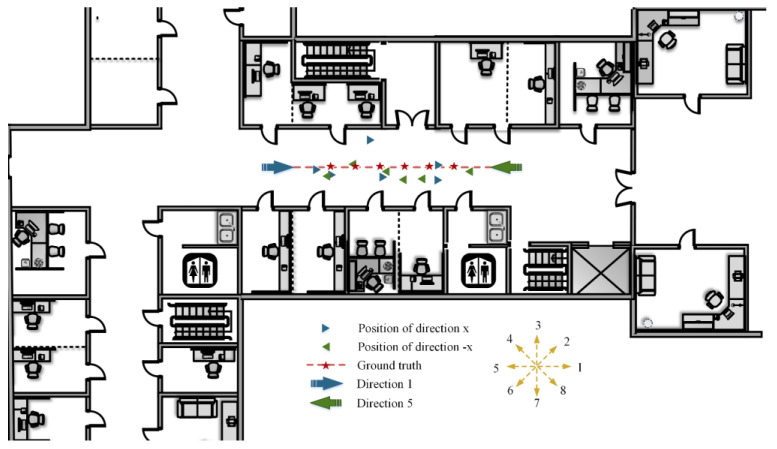
The positioning results of the fixed points when the user walks in different directions.

In order to verify the actual positioning performance of the WeChat mini program and the localization method proposed in this paper, a positioning experiment for a user was conducted in scene Ⅱ to simulate the real situation. The corresponding positioning results are shown in Figure 30.

In the experiment, we walked along the central red dotted line in the corridor in directions 1 and 5. Then, we stopped, uploaded a clear photo taken at each asterisk location, and obtained the positioning result. The asterisk positions are the pre-marked fixed points. The locations of the blue and green triangles correspond to the results according to different images obtained in directions 1 and 5, respectively. It can be seen that the method proposed in this paper can acquire an accurate location without additional equipment, meet the application requirements of the actual indoor environment for user positioning, and achieve the effect of real time and practicability.

## 5. Discussion

Whether using a linear algorithm or a nonlinear algorithm to estimate the fundamental matrix, the core idea is to transform the problem into an optimization problem. When there are many matching points, the computational complexity of this method is large, and it is difficult to obtain a globally optimal solution. Therefore, how to design the solution method of the fundamental matrix and further improve the accuracy and stability of the fundamental matrix estimation will be a follow-up research direction.

## 6. Conclusions

This paper studied the visual positioning of large indoor places such as teaching buildings, hospitals, libraries, shopping malls, and parking lots. Both ICP and PnP need to obtain the actual 3D coordinates of some feature points in space to achieve 3D–3D and 3D–2D position estimation. Considering factors such as ease of use, equipment price, and deployment cost, it is more cost-effective to obtain location information from 2D images. However, the existing methods to calculate the positioning coordinates using the pose obtained from the epipolar geometry project the 3D vector onto the 2D plane and select the intersection point, which may be at infinity as a result. Therefore, we proposed an indoor visual positioning method with 3D coordinates using an accelerometer and magnetometer to realize the precise positioning of indoor users.

Firstly, the checkerboard calibration board was established, and the internal parameters satisfying the reprojection error were obtained according to Zhang’s calibration method for fundamental matrix calculation. We constructed an offline dataset in two scenes and marked the pose obtained by built-in smartphone sensors and the position acquired by a laser rangefinder onto the images taken at corresponding locations. Secondly, 3D RLM-TDS was designed to transform the positioning problem into solving the minimum distance from one point in the space to multiple straight lines. Experiments were carried out to determine the optimal threshold of the constraint method so as to limit the location of the positioning results. Thirdly, the localization experiment and result visualization were carried out in three situations of different range images as the retrieval results, with different camera poses and different feature extraction methods. The findings indicated that, even when the retrieval results are the worst, the positioning method could still achieve 90% positioning effect with an error of less than 0.58 m under different poses. Moreover, the method was not limited to a single feature extraction method, and the average positioning error was lower than 0.32 m. Lastly, a WeChat mini program mounted on mobile devices was designed to realize dynamic experiments, and the positioning method proposed in this paper was compared with other recent work. The results showed that the proposed 3D RLM-TDS achieves ease of use under the condition of low equipment and deployment cost, while meeting the error requirements of user positioning in practical applications.

## Figures and Tables

**Table 1:** Camera calibration parameters.

Camera Parameters	Specific Parameters	Values
Intrinsic parameters	Focal length	[563.1549 561.96807]
	Principal point	[457.5447 344.0597]
Lens distortion	Radial distortion	[0.2042 −0.9537 1.0331]
	Tangential distortion	[2.6321 × 10^−4^–4.0797 × 10^−4^]
Accuracy of estimation	Mean reprojection error	0.2380

**Table 2:** Localization accuracy and improvement rate under different ranges of retrieval results.

Scope of Source	Distance between Retrieved Images and Query Image	Maximum Distance between Retrieved Images	90% 2D Positioning Error (m)	Improvement Rate of 2D Positioning Error	90% 3D Positioning Error (m)	Improvement Rate of 3D Positioning Error
4 points	0.60	1.20	0.27	55.00%	0.32	46.67%
8 points	0.85	1.70	0.54	36.47	0.54	36.47%
10 points	1.20	2.40	0.62	48.33%	0.63	47.50%
14 points	1.34	2.68	0.79	41.04%	0.79	41.04%

**Table 3:** Conversion of the camera coordinate system and world coordinate system under the condition of each pose.

Pose	Angle	Rotation Matrix	Pose	Angle	Rotation Matrix
Pose 1	*y*: −90° *x*: 90°	$\left[\begin{array}{ccc} 0 & 0 & 1\\ -1 & 0 & 0\\ 0 & -1 & 0 \end{array}\right]$	Pose 2	*y*: −45° *x*: 90°	$\left[\begin{array}{ccc} 0.707106781 & 0 & 0.707106781\\ -0.707106781 & 0 & 0.707106781\\ 0 & 1 & 0 \end{array}\right]$
Pose 3	*y*: 0° *x*: 90°	$\left[\begin{array}{ccc} 1 & 0 & 0\\ 0 & 0 & 1\\ 0 & -1 & 0 \end{array}\right]$	Pose 3	*y*: 45° *x*:90°	$\left[\begin{array}{ccc} 0.707106781 & 0 & -0.707106781\\ 0.707106781 & 0 & 0.707106781\\ 0 & -1 & 0 \end{array}\right]$
Pose 5	*y*: 90° *x*: 90°	$\left[\begin{array}{ccc} 0 & 0 & -1\\ 1 & 0 & 0\\ 0 & -1 & 0 \end{array}\right]$	Pose 6	*y*: 135° *x*: 90°	$\left[\begin{array}{ccc} -0.707106781 & 0 & -0.707106781\\ 0.707106781 & 0 & -0.707106781\\ 0 & -1 & 0 \end{array}\right]$
Pose 7	*y*: 180° *x*: 90°	$\left[\begin{array}{ccc} -1 & 0 & 0\\ 0 & 0 & -1\\ 0 & -1 & 0 \end{array}\right]$	Pose 8	*y*: −135° *x*: 90°	$\left[\begin{array}{ccc} -0.707106781 & 0 & 0.707106781\\ -0.707106781 & 0 & -0.707106781\\ 0 & -1 & 0 \end{array}\right]$

**Table 4:** Comparison of average error of different positioning methods. The check and cross marks indicate whether 3D coordinates can be obtained and whether there are large outliers.

Position Determination	Pose Acquisition	3D Coordinate	Large Outlier	Average Positioning Error (m)
ORB image retrieve	×	√	×	1.887250
SURF image retrieve	×	√	×	0.724250
N × dis [34]	LMedS	×	√	1.431490
N × dis [34]	RANSAC	×	×	1.335285
Dis [32,33]	LMedS	×	√	1.062031
Dis [32,33]	RANSAC	×	×	0.877582
3D RML	LMedS	√	×	0.539870
3D RML	RANSAC	√	×	0.487767
3D RML-TDS	LMedS	√	×	0.318551
3D RML-TDS	RANSAC	√	×	0.315481

## Data Availability

The data presented in this study are available on request from the corresponding author. The data are not publicly available due to privacy.
